# A Computational Study of Metallacycles Formed by Pyrazolate Ligands and the Coinage Metals M = Cu(I), Ag(I) and Au(I): (pzM)*_n_* for *n* = 2, 3, 4, 5 and 6. Comparison with Structures Reported in the Cambridge Crystallographic Data Center (CCDC)

**DOI:** 10.3390/molecules25215108

**Published:** 2020-11-03

**Authors:** José Elguero, Ibon Alkorta

**Affiliations:** Instituto de Química Médica (IQM-CSIC), Juan de la Cierva, 3, E-28006 Madrid, Spain; iqmbe17@iqm.csic.es

**Keywords:** X-ray, pyrazolate, coinage metals, metallacycles, M06-2x

## Abstract

The structures reported in the Cambridge Structural Database (CSD) for neutral metallacycles formed by coinage metals in their valence (I) (cations) and pyrazolate anions were examined. Depending on the metal, dimers and trimers are the most common but some larger rings have also been reported, although some of the larger structures are not devoid of ambiguity. M06-2x calculations were carried out on simplified structures (without C-substituents on the pyrazolate rings) in order to facilitate a comparison with the reported X-ray structures (geometries and energies). The problems of stability of the different ring sizes were also analyzed.

## 1. Introduction

There is a field in organometallic chemistry that has rightly demanded a great deal of attention, namely, the cyclic complexes between coinage metal cations and anionic pyrazolate ligands [1]. These metallacycles frequently have high symmetry and contain several nuclei with spin I = 1/2, which makes them ideally suited for NMR studies: ^1^H, ^13^C, ^15^N, ^19^F (from the much studied 3,5-bis-trifluoromethylpyrazole) and ^107/109^Ag. In contrast, ^63/65^Cu and ^197^Au are quadrupolar (I = 3/2) and have therefore been explored to a much lesser extent.

It is common in publications in the field of coordination compounds that single-crystal X-ray structures are reported. In such cases the data are in the Results and Supporting Information sections. Note that there are some publications that concern other aspects of these compounds such as their use in sensors, optical properties, and theoretical calculations where crystal structures are not reported. Earlier papers should also be mentioned here because, although they do not contain crystal structures, they were key in generating interest in these metallacycles [2,3,4]. Lintang et al. reported that trinuclear group 11 metal pyrazolate complexes are phosphorescent chemosensors for the detection of benzene [5] (for two recent papers on photoluminescence of the Dias and Fujisawa groups see [6,7]). The Serrano group published several papers that describe compounds related to those discussed in the present paper but with interesting mesogen properties when the pyrazolate ligands have long chains in positions three and five and the metal is Cu, Ag or Au [8,9,10,11]. Cano’s group published similar results for complexes with gold(I) [12].

Of particular relevance is a theoretical paper concerning the study of group 11 pyrazolate complexes. In this case, Caramori, Frenking et al. [13] discussed the trinuclear (pzM)_3_ complexes (M = Cu(I), Ag(I) and Au(I)) in terms of different approaches including energy decomposition analysis (EDA), natural bond orbital (NBO) and anisotropy of the induced current density (ACID). The main conclusions were that the pz-M bond has an elevated covalent character, especially when M = Au(I), and that the pyrazole ligands are strongly aromatic, although they are insulated because there is no through-bond metal-ligand conjugation.

Our group published a paper, in collaboration with Rasika Dias and another with Kiyoshi Fujisawa, on the NMR study of the organometallic nine-membered rings corresponding to trinuclear silver(I) complexes of pyrazolate ligands [14,15], and another on regium bonds between dinuclear silver(I) pyrazolates complexes and Lewis bases [16], two on regium bonds formed by Au_2_ [17] and Ag_2_, Cu_2_ and mixed binary regium molecules [18], and finally, one on the comparison of acidity of Au(I) and Au(III) [19].

## 2. Results and Discussion

The present publication is divided into three sections. The first section concerns an exploration of the Cambridge Structural Database (CSD) [20] in a search for the structures of pyrazolates with coinage metals of valence (I): i.e., Cu(I), Ag(I) and Au(I); they will be reported using their refcodes. The second section covers a theoretical study of the stability of these metallacycles as a function of the ring size (dimers, trimers, tetramers, pentamers and hexamers) using the pyrazole itself as a model, i.e., without C-substituents and without supplementary ligands on the metals. The final section concerns the analysis of some metallacycles by Bader’s quantum theory of atoms in molecules methodology (QTAIM) [21,22,23,24].

**Figure 1:**
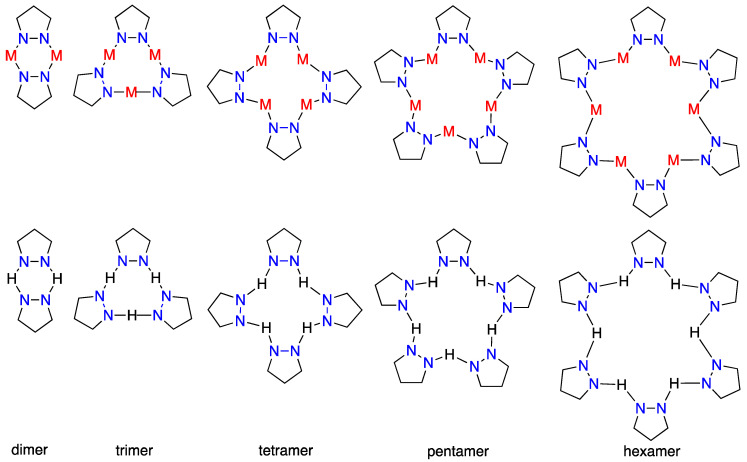
Metallacycles (**top**) and the corresponding cyclamers (**bottom**).

We will start with the exploration of the CSD [20]; this search was similar to one carried out by us on the cyclamers formed by NH-pyrazoles based on hydrogen bonds (HBs). NH-pyrazoles crystallize as catemers (chains) and cyclamers (rings with *n* pyrazoles), with examples reported for *n* = 2, 3, 4, and 6 (rare) but none for a pentamer [25,26] (Figure 1).

### 2.1. Analysis of the Reported CSD Structures (Hits) and Their Refcodes

**Figure 2:**
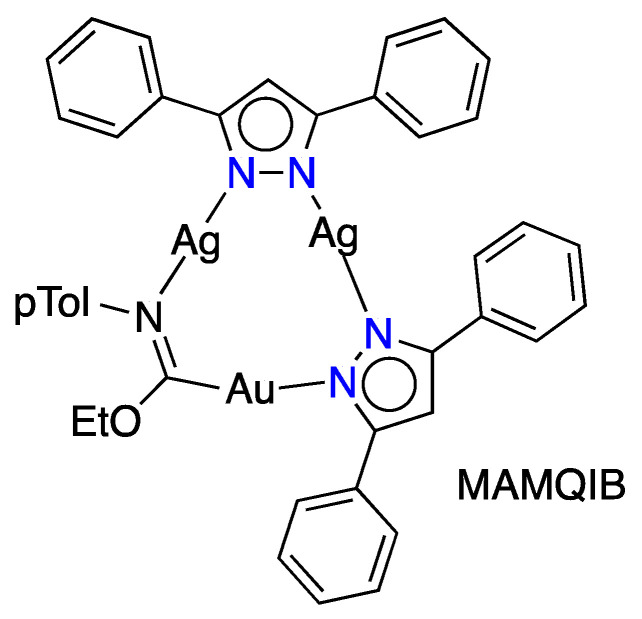
Structure of CSD Refcode MAMQIB.

Before discussing the most relevant hits, it is worth noting that there is only one compound that contains two different metals (Ag_2_Au), namely a gold(I)imidazolate-silver(I)pyrazolate complex, MAMQIB [27] and MAMQIB01 [28] (Figure 2), but this only has two pyrazolates, with the third ligand being an carbeniate (C,N ligand). In any case, this example shows that [pzM(M′)]*_n_* compounds should be possible with different metals, although examples have not been reported to date.

#### 2.1.1. Copper, Only Cu(I) Structures (Cu(II) Structures Were Excluded)

**Figure 3:**
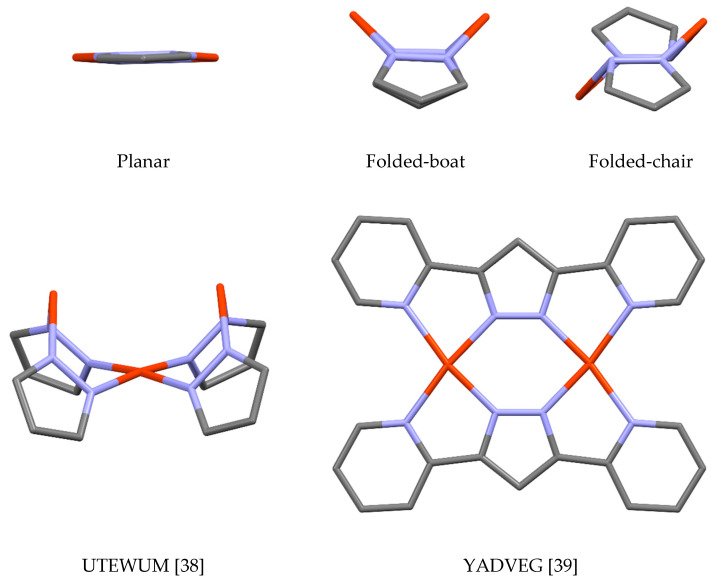
Planar and folded conformations of the metallacycle in (pzCu)_2_ (simplified representations of UTEWUM and YADVEG).

Dimers and trimers, (pzCu)_2_ and (pzCu)_3_, are common. As far as dimers are concerned, many of these are Cu(II) derivatives and all examples that contain Cu(I) have complex pyrazolates with arms that are able to coordinate the copper or they have supplementary ligands; dimers in which the Cu(I) atoms are “nude”, i.e., linked only to the pyrazolate anion, have not been found. Amongst the supplementary ligands are CO (COCZAY [29], COCZOM [29]), C≡N–R (GITJUO [30], HEDFIF [31], JEMCAF [32]), PPh_3_ (GITKEZ [30], PIRDAY [33], SATKAC [34]), pyridines (IPIGET [35], KUKLOR [36]) and *N*-heterocyclic carbenes NHCs (NETLUW [37], NETMAD [37]). The central metallacycle can adopt planar or folded conformations, either boat-type or chair-type (Figure 3), and both of these conformations are common. An examination of the Cu···Cu distances in (pzCu)_2_ structures gave 320 values, with a mean value of 3.719 Å, a minimum of 3.013 Å (UTEWUM [38]) and a maximum of 4.074 Å (YADVEG [39]) (Figure 3).

As far as trimers are concerned, the two main classes are isolated trimers and double trimers and we proposed to denote the latter as “3 + 3”. The simplest derivatives of isolated trimers include CODBAB (4-Cl-pyrazolate) [40], VIJMUW (3,5-diMe-pyrazolate) [41], BELTOC (3,5-di-*i*-Pr-pyrazolate) [42] and XELXAN (3,5-di-CF_3_-pyrazolate) [43].

**Figure 4:**
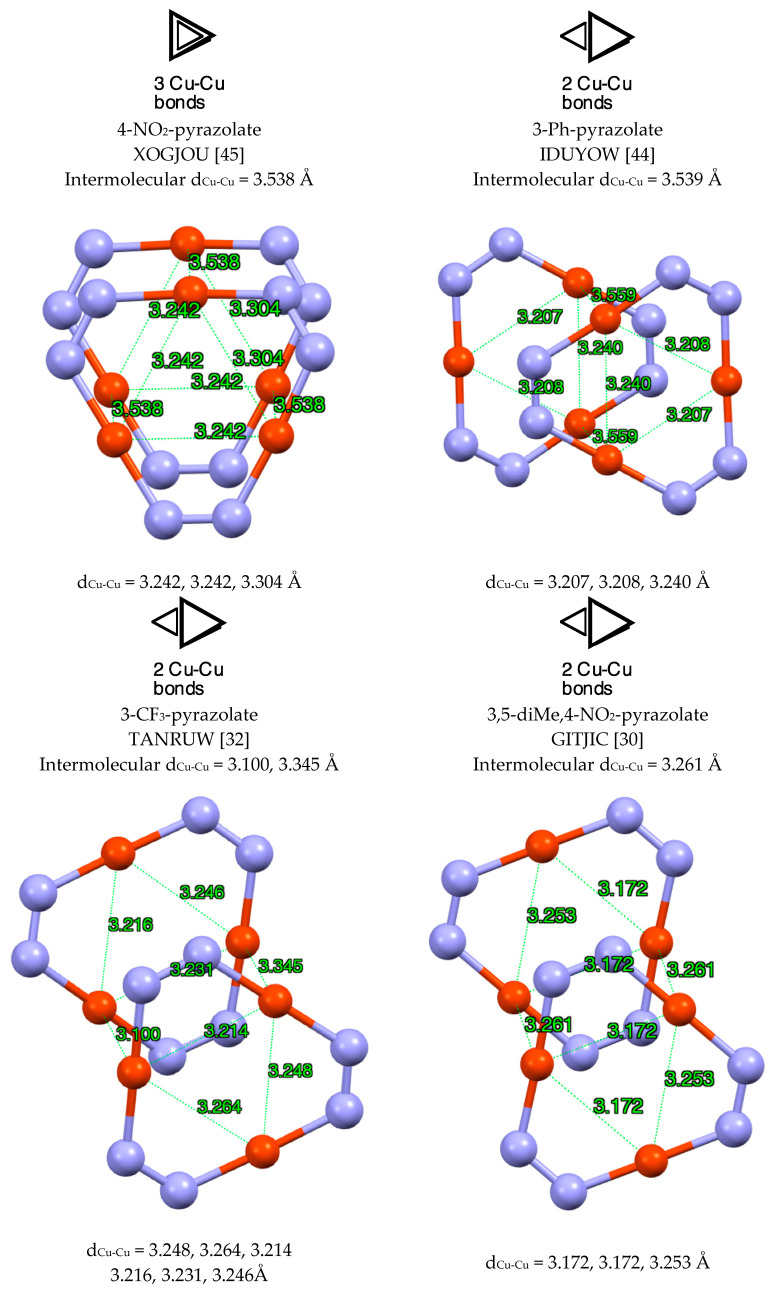
Different modes present in the “3 + 3” (pzCu)_3_ complexes. Only the copper and nitrogen atoms are represented.

Simple examples of “3 + 3” double trimers (Figure 4) are IDUYOW (3-Ph-pyrazolate) [44], TANRUW (3-CF_3_-pyrazolate) [32] and GITJIC (3,5-diMe-4-NO_2_-pyrazolate) [30] with near one bond between the triangles formed by Cu atoms and XOGJOU (4-NO_2_-pyrazolate) [45] with near three Cu-Cu bonds. The superimposed structures of double trimers can be a perfect fit, e.g., XOGJOU [45] or rotated (an example will be discussed later for another metal).

**Figure 5:**
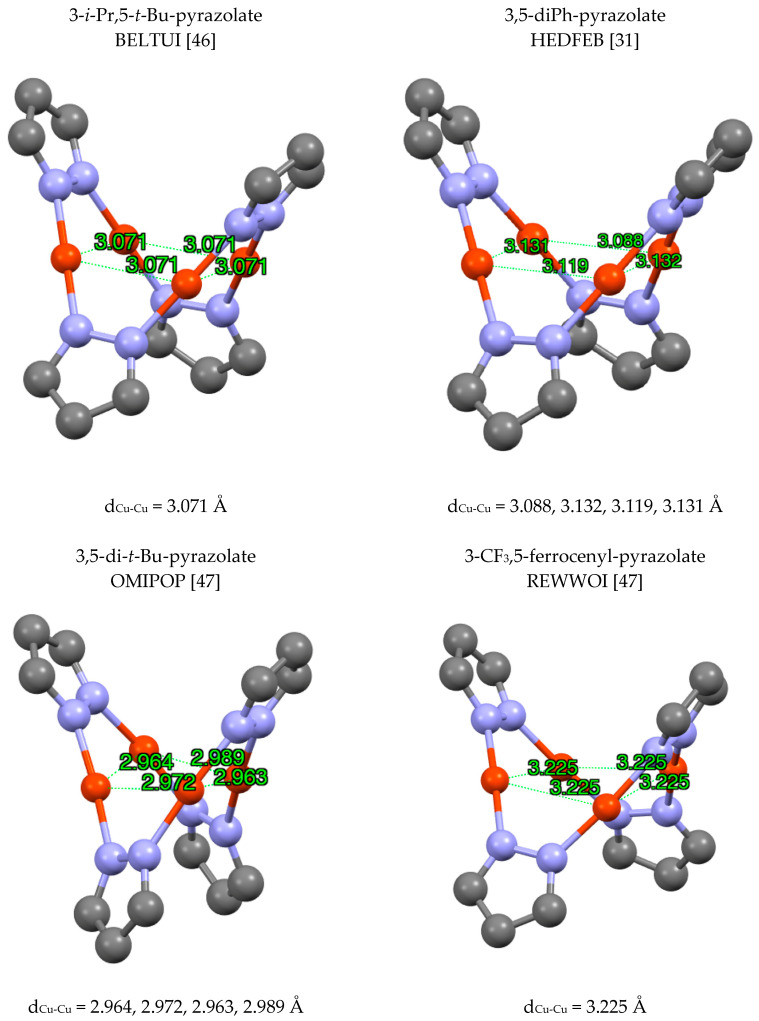
Different modes of (pzCu)_4_ complexes. Only the copper atoms and the pyrazolate rings are represented. The sequential Cu-Cu distances are listed.

Tetramers (pzCu)_4_ are frequently observed and the simplest derivatives include BELTUI [42] (and BELTUI01 [46]), HEDFEB [31], (and OMIPOP01 [42]), OMIPOP [47] and REWWOI [48] (Figure 5). Most Cu_4_ rings are planar but in REWWOI [48] this ring is not planar. The structure of OMIPOP [42] was represented with two Cu atoms bonded to both N atoms of the corresponding pyrazole but this is only the result of a CSD convention that bonds are depicted when they are shorter than the sum of the van der Waals radii. In the original article [48] it is highlighted that the four Cu atoms form a rhombus with a Cu···Cu non-bonding interaction for the shortest distance corresponding to d^10^-d^10^ contacts. Fujisawa re-examined this interesting structure (OMIPOO01 [42] and OMIPOO02 [46]) and noted the diamond-like disposition of the four Cu atoms with a short (3.40 Å) and a long (4.85 Å) structure.

At this point it is worth commenting on hexamers, (pzCu)_6_; these structures always contain six central OH bridges to form a star of alternated Cu atom and O atoms, which means that these are Cu(II) derivatives (QIMWOA and QIMWUG (Coronado et al. [49]) and SASXIW (Galassi, Martins et al. [34])). In summary, examples of Cu(I) pentamers or hexamers are not known.

#### 2.1.2. Silver, Only Ag(I) Derivatives

Compounds with (pzAg)_2_ and (pzAg)_3_ structures are common. As in the case of (pzCu)_2_, in (pzAg)_2_ the hexagonal metallacycle adopts planar and folded conformations, with the latter being either boat-likes (the most common) or chair-like (as in cyclohexane), with the silver atoms located at the tips. The mean value of the Ag···Ag distance is 3.755 Å (shortest 3.425 Å, longest 4.305 Å).

**Figure 6:**
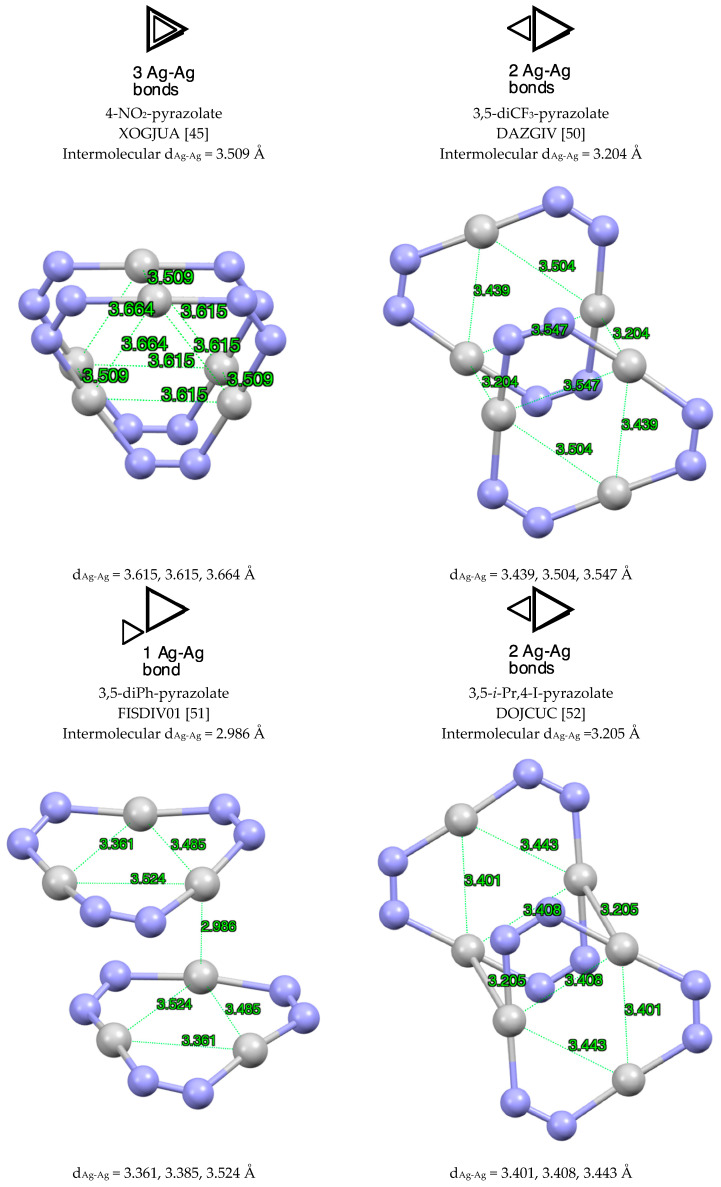
Different modes of “3 + 3” (pzAg)_3_ complexes. Only the silver and nitrogen atoms are represented.

Three topological dispositions of double trimers “3 + 3” were found in the CSD (Figure 6), namely three common sides, one common side and one common vertex. These structures are schematically represented in Figure 6 with triangles. These dispositions are illustrated with one or two examples for each situation: XOGJUA [45], DAZGIV [50], FISDIV01 [51] and DOJCUC [52] (EWEHAP [53] is similar with an intermolecular distance between silver metal centers of 3.179 Å). The intermolecular Ag···Ag distance decreases with the number of bonds (3.509 Å (three), 3.205 Å (two) and 2.986 Å (one)) and this is probably due to angular strain.

**Figure 7:**
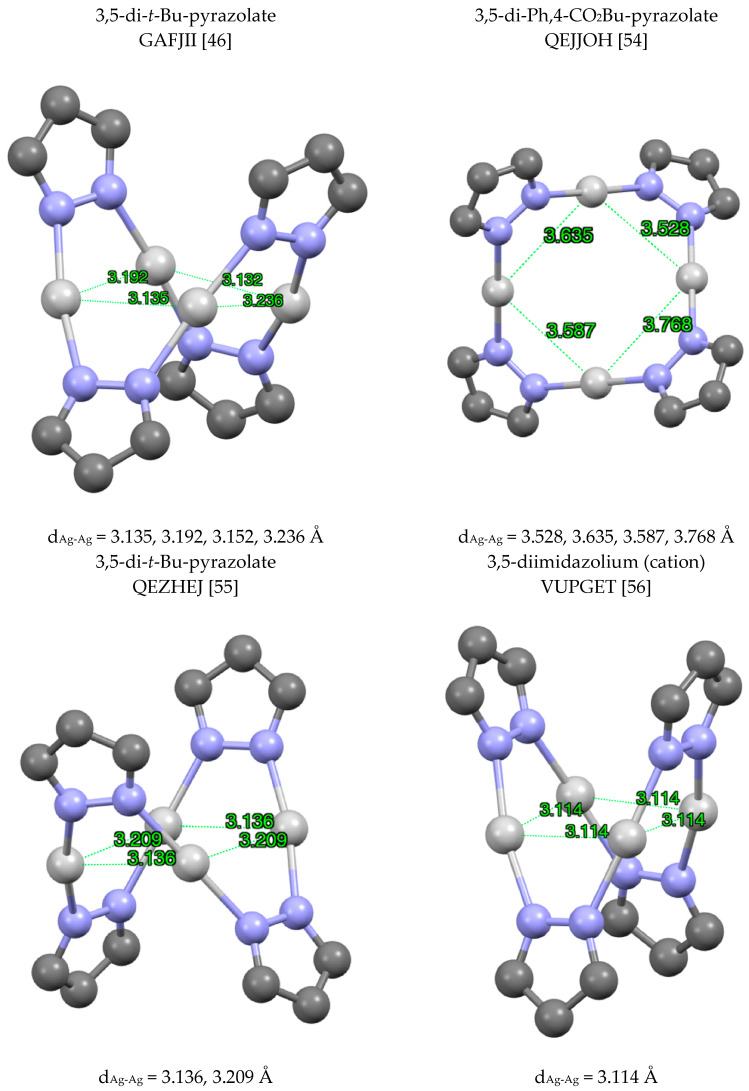
Different structures of (pzAg)_4_ complexes. Only the silver atoms and the pyrazolate rings are represented. The four examples are very similar but different views have been used.

Tetramers (pzAg)_4_ are frequent and, in this case, the simplest structures correspond to GAFJII [46], QEJJOH [54], QEZHEJ [55] and VUPGET [56] (Figure 7).

**Figure 8:**
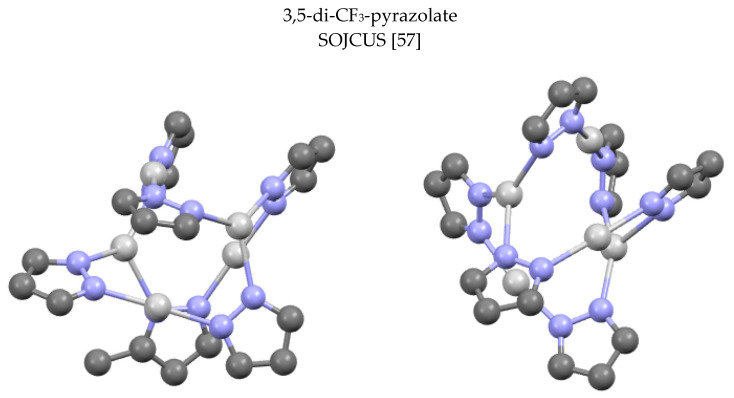
A view of the two independent molecules of SOJCUS. Only the silver atoms and the pyrazolate rings are represented.

The structure of SOJCUS [57] is rather complex (Figure 8) in that the silver atoms are double-, triple- and quadruple-coordinated, thus allowing a structure that contains six pyrazolates and five silver atoms. Depending on the itinerary four, (pzAg)_4_, and five, (pzAg)_5_, complexes are present (Figure 8), but a true tetramer or pentamer does not exist.

**Figure 9:**
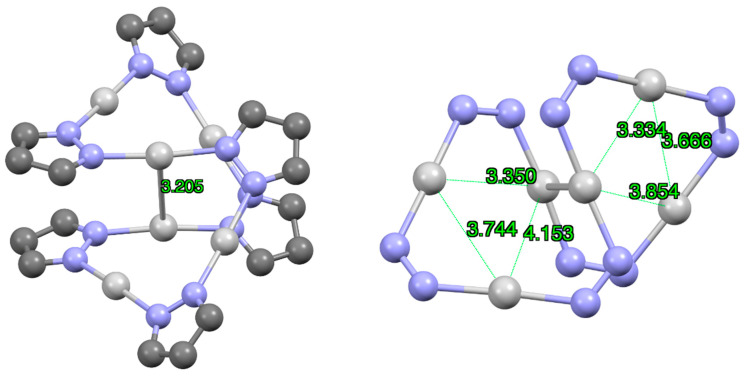
Two views of QEJJEX. Only the silver atoms and the pyrazolate rings are represented. Left Ag···Ag distance between loops; right Ag···Ag distance in the quasi-triangles.

The only examples of hexamers are QEJJEX [54] and QEJJIB [54] (almost identical), which are beautiful structures that form a loop with short Ag···Ag contacts (QEJJEX [54], Figure 9).

In the case of silver, the numbers of structures found in the CSD search were 72 trimers, 16 dimers, 6 tetramers and 2 hexamers (2.1%) (pentamers were not found).

#### 2.1.3. Gold, Only Au(I) Structures (Au(III) Structures Were Excluded)

**Figure 10:**
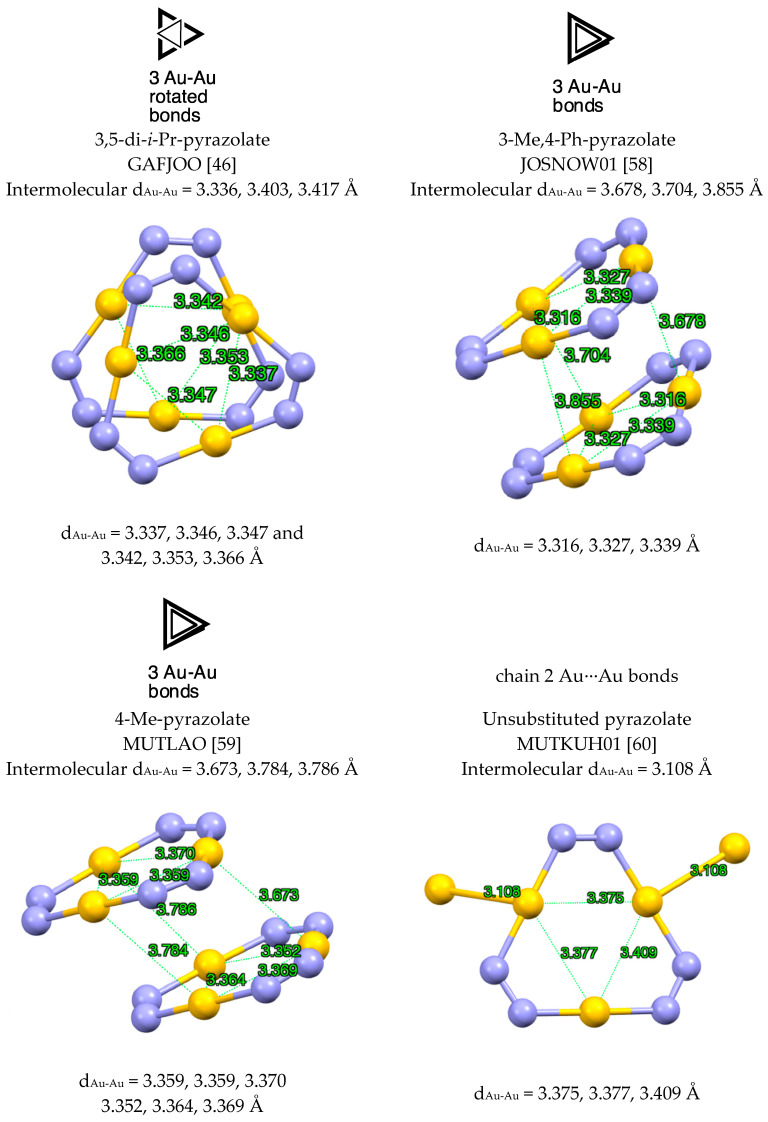
Different modes of (pzAu)_3_ “3 + 3” complexes. Only the gold and nitrogen atoms are represented [59,60].

Dimers (pzAu)_2_ with Au(I) are not known and all of the examples found in the CSD are Au(III) derivatives. Trimers (pzAu)_3_ are common, with some compounds isolated as trimers, e.g., the bis-3,5-CF_3_-pyrazolate, COHFIO01 [50] (d_Au-Au_ = 3.341, 3.350, 3.360 Å) and bis-3,5-Ph-pyrazolate FUWXOK01 [58] (d_Au-Au_ = 3.361 Å, a regular triangle) while others (Figure 10) are “3 + 3” double trimers.

**Figure 11:**
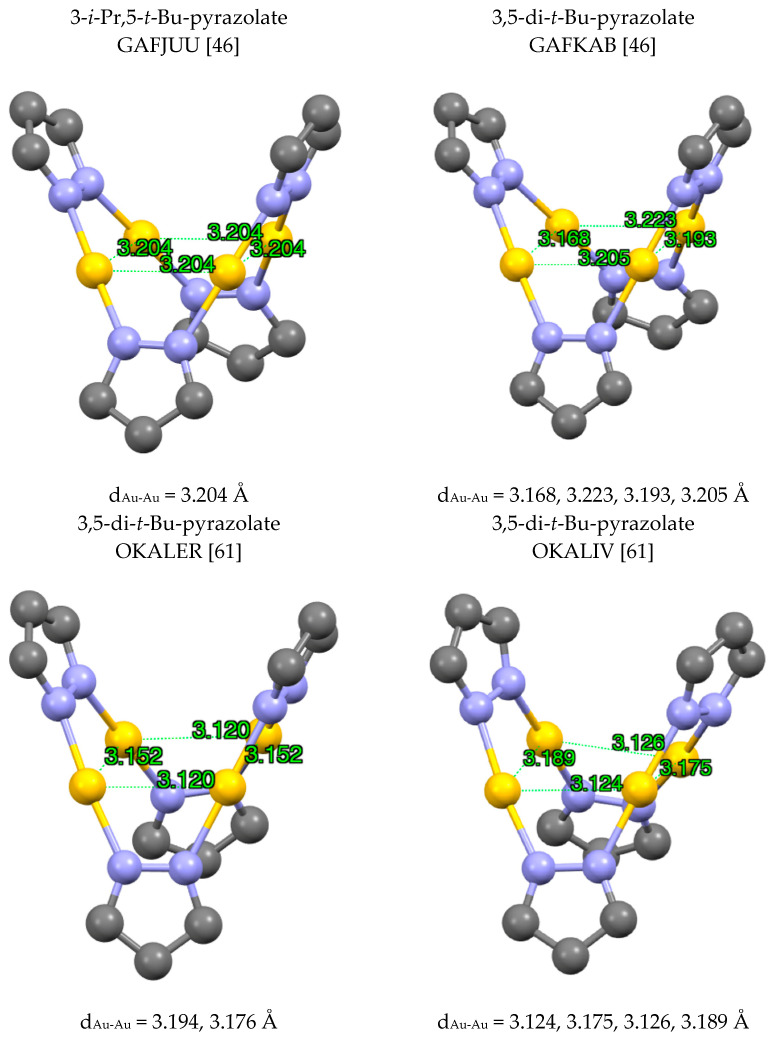
Different modes of (pzAu)_4_ complexes. Only the gold atoms and the pyrazolate rings are represented.

Au(I) tetramers, (pzAu)_4_, are known and the simplest examples are GAFJUU [46], GAFKAB [46], OKALER [61] and OKALIV [61] (Figure 11). The gold centers can arrange in a regular square (GAFJUU [46]), in a rectangle (OKALER [61]), in a quadrilateral (GAFKAB [46]) or in a folded quadrilateral (OKALIV [61]).

**Figure 12:**
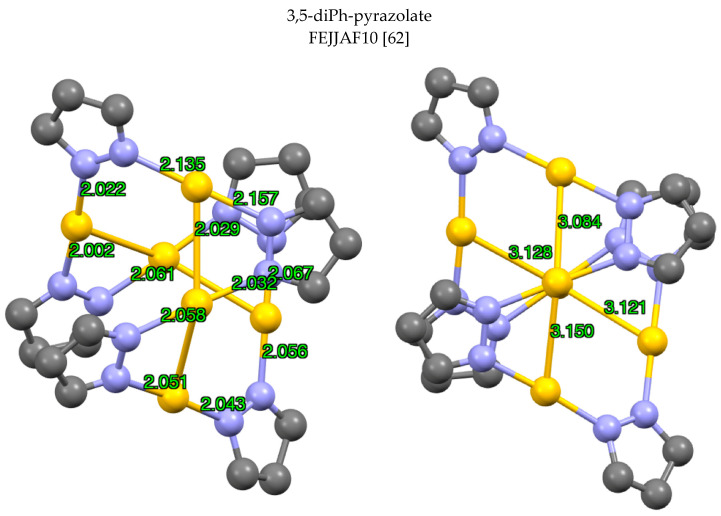
Two views of one of the two independent molecules of FEJJAF10 with Au-N distances (**left**) and Au-Au distances in Å (**right**). Only the gold atoms and the pyrazolate rings are represented.

Finally (pzAu)_6_ structures are very rare: in 1988 Raptis reported FEJJAF10 [62], see Figure 12.

On examining the structures of gold(I) reported in the CSD we found 28 trimers, 8 tetramers and 1 hexamer (dimers and pentamers were not found).

The X-ray structures previously discussed are summarized in Table 1 together with NH-pyrazole cyclamers.

Thus, the situation has some similarities for H and for M in the sense that NH pyrazoles cyclamers with *n* = 2, 3, 4, and 6 (rare) have been reported but a pentamer (**5**) has not been reported [25,26]. However, while metallacycles trimers are the most common (**1**), in NH-pyrazoles they occupy only the third position (**3**).

### 2.2. Calculated Structures (Minima in All Cases)

#### 2.2.1. Geometries

**Figure 13:**
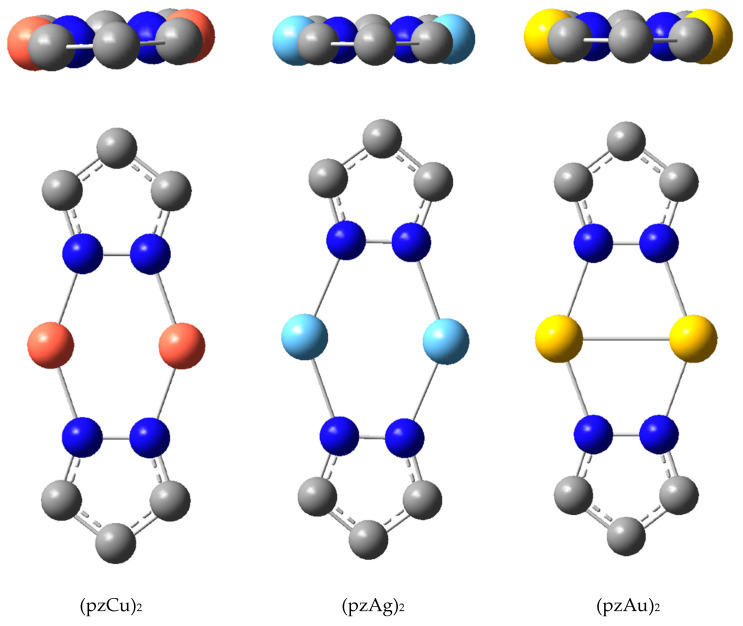
Two views of the calculated Cu, Ag and Au dimers; hydrogen atoms have been omitted.

We calculated different dispositions of the metallacycles using the parent pyrazolate ligand as a model, i.e., without any C-substituent. In the case of dimers, all adopt the planar conformation and never the folded conformation found in the CSD (Figure 13 and Table 2).

**Figure 14:**
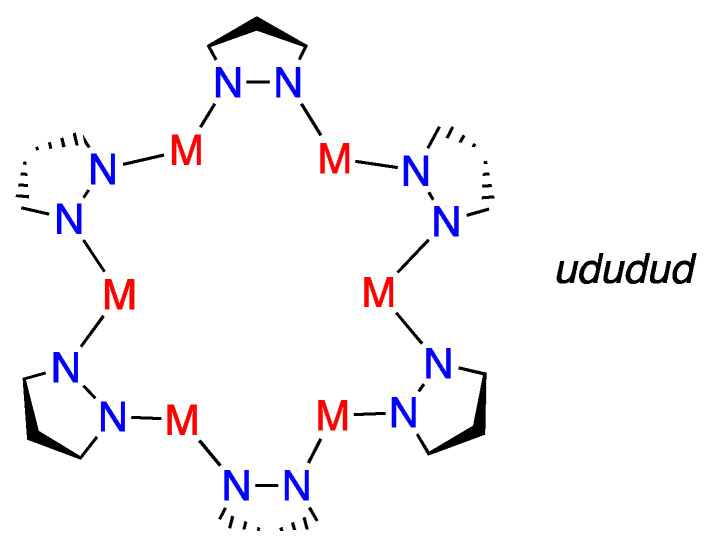
Schematic representation of the *ududud* structure.

The trimers lead to triangles and it is interesting to estimate how far they are from the equilateral case that results from a *D_3h_* symmetry in the examples reported in the CSD. The tetramers will lead to squares (*D_4h_*), planar deformed squares (rectangles, rhombs) and non-planar structures (folded about the M1–M3 edge). The situation increases in complexity as the number of metals increases; for hexamers there are the planar regular hexagon (*D_6h_*) and several distorted hexagons, including the *ududud* structure (*u* or *d* refers to the *up* or *down* position of the pyrazole ring, as shown schematically in Figure 14).

We also calculated double dimers (2 + 2) and double trimers (3 + 3) in two orientations. The structures and some distances are represented in the following images for all metallacycles except dimers. The distances that were analyzed are M(I)···M(I) and N···M(I).

**Figure 15:**
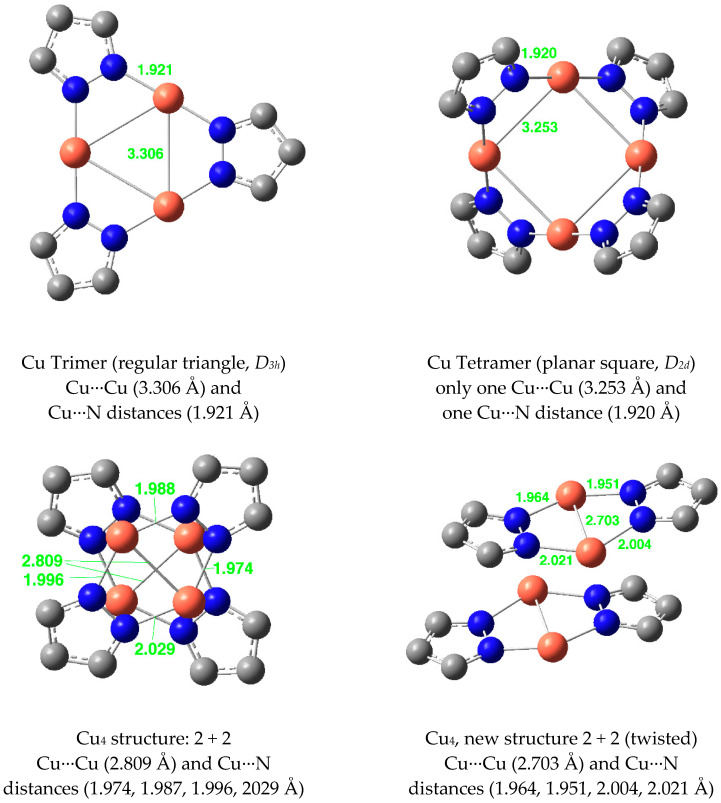

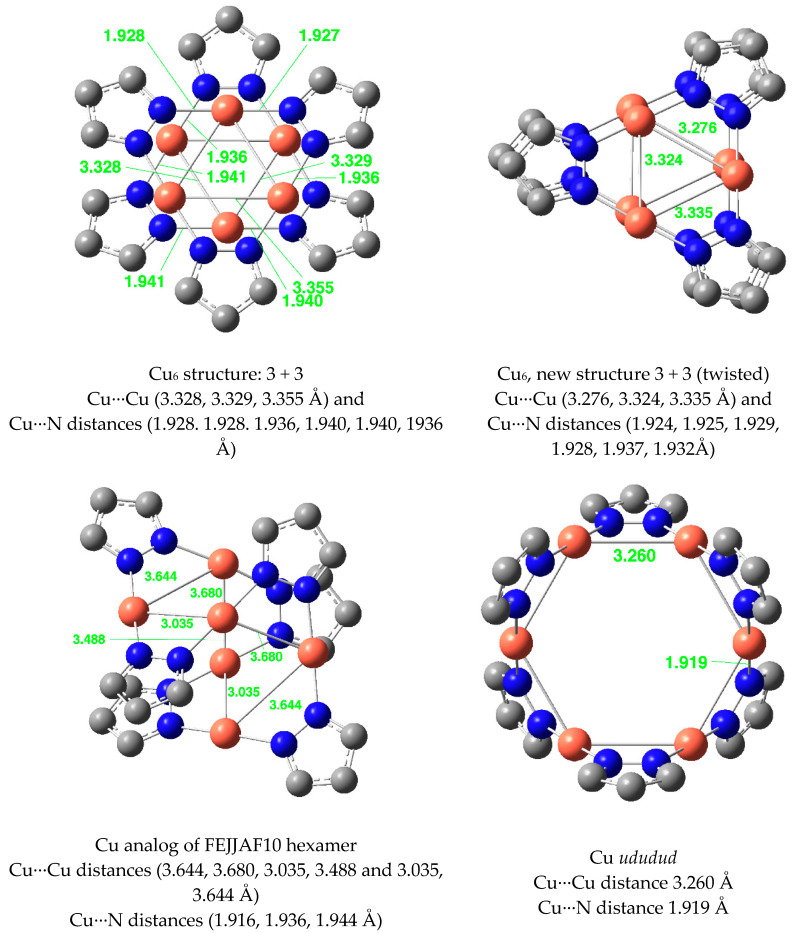
Optimized structures of Cu(I) pyrazolates.

The studied Cu(I) derivatives, beyond monomers and dimers, are represented in Figure 15. The mean distances in the dimers are Cu···Cu = 2.656 Å and N···Cu = 1.963 Å.

**Figure 16:**
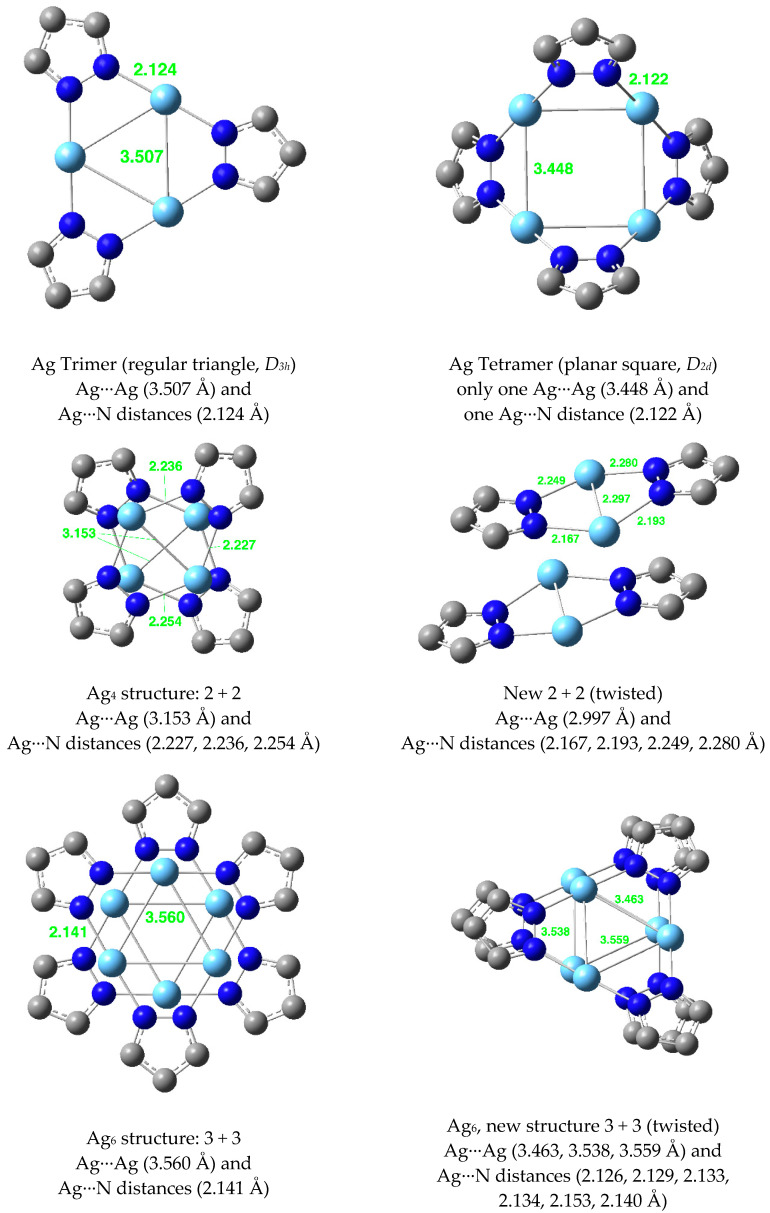

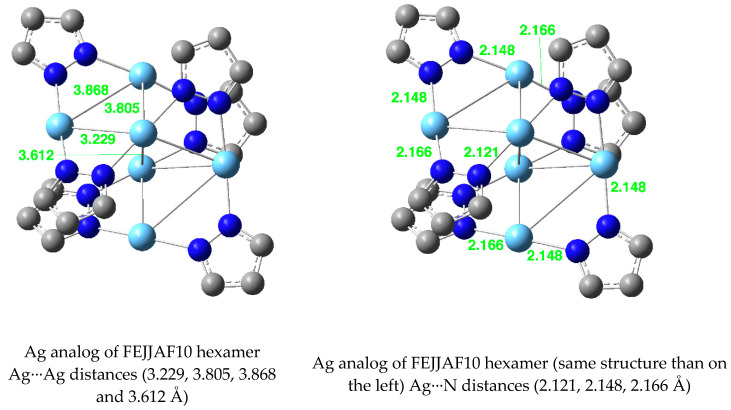
Optimized structures of Ag(I) pyrazolates.

The Ag(I) structures are reported in Figure 16. The average distances for the dimer are Ag···Ag = 2.953 Å and N···Ag = 2.192 Å.

**Figure 17:**
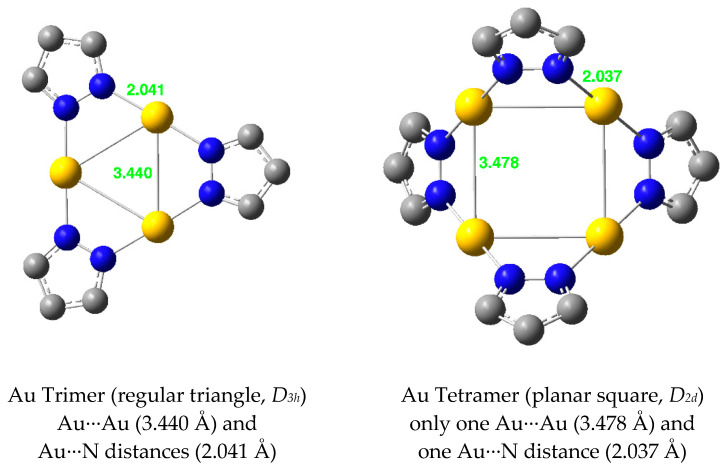

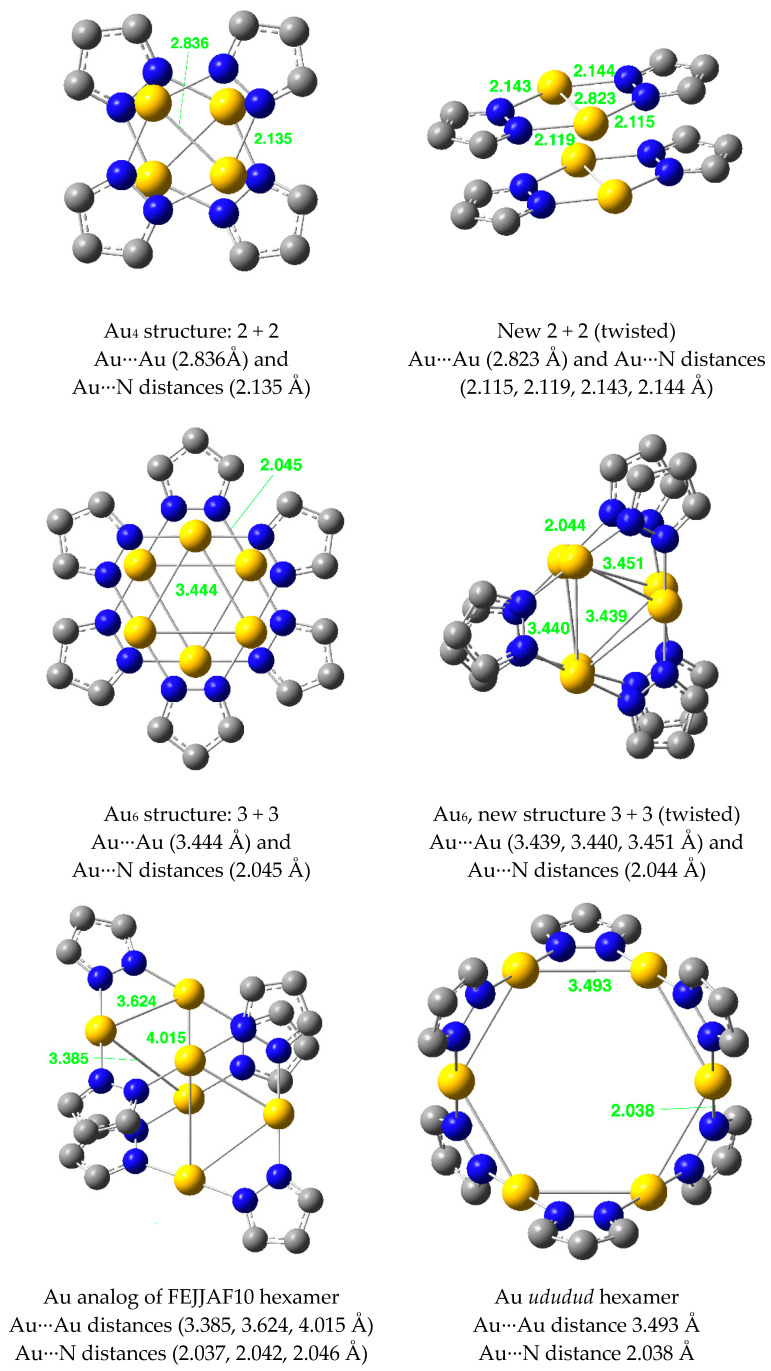
Optimized structures of Au(I) pyrazolates.

Finally, the studied Au(I) derivatives are represented in Figure 17. For the dimer: Au···Au = 2.808 Å and N···Au = 2.124 Å.

#### 2.2.2. Comparison of Calculated and Measured Geometries (Only Metal···Metal and Metal···Nitrogen Bond Lengths)

The average metal-metal and metal-nitrogen bond lengths in our calculations and in the structures found in the CSD search are gathered in Table 2, Table 3 and Table 4.

A statistical analysis of the results in Table 2, Table 3 and Table 4 provided the following three equations:

Averaged = (0.98 ± 0.01) Parent, *n* = 12, *R*^2^ = 0.998, RMS residual = 0.12 Å (1)

Averaged = (0.96 ± 0.01) + (0.93 ± 0.09) dimer, *n* = 20, *R*^2^ = 0.998, RMS residual = 0.12 Å (2)

Parent = (1.00 ± 0.01) + (0.96 ± 0.06) dimer, *n* = 12, *R*^2^ = 0.999, RMS residual = 0.08 Å (3)

Equation (1) shows that averaged and parent pyrazole values are roughly proportional with a slope of 0.98 indicating that the averaged values are slightly smaller than the parent ones.

Equations (2) and (3) are similar, while (2) is better than (3), with a slope = 1.00 indicating that our calculated geometries that correspond to pyrazole itself are closer to a model of “parent” pyrazoles. It was found in a previous study [16] that the Ag···Ag distances of (pzAg)_2_ are very sensitive to the ancillary ligands. If we assume that the situation is the same for the Cu(I) ligands (there are no examples of Au(I) dimers) it is sufficient to add a term (a dummy variable, one if dimers, zero if other metallacycles). The result is 0.93–0.96 Å and this indicates that the contraction of the Ag···Ag distance due to ancillary ligands is very important.

### 2.3. Energies

We start with a very simple premise that the more abundant a metallacycle of a given size found in the CSD the more stable the structure. A step further is to consider the percentages as a quantitative measurement of the stability in a sort of Maxwell-Boltzmann distribution. This implies two things: that the number of examples is very large and that the structures are in equilibrium (thermodynamic control). Clearly these conditions are not fulfilled, but it remains interesting to explore the possibility of partial agreement. In this work we explored the ring size, in NH-pyrazole cyclamers we successfully studied the effect of the C-substituents [25,26] and, finally, in the case of Ag(I) pyrazolate dimers we studied the effect of ancillary ligands, which can have a marked effect on the Ag···Ag distance with a concomitant decrease in stability that was compensated for by the ligands [16]. Consequently, the problem is of great complexity and it is useful to remember that the mechanism of crystal growth is also complex and is not fully understood [72,73].

In an effort to compare the stabilities of the different metallacycles we calculated their relative free energies, ΔG_rel_ in kJ mol^−1^, per metallacycle and per monomer. The results are provided in Table 5. *δ*ΔG_rel_ = [ΔG_rel_ − ΔG_rel_ (minimum)] × *n*. The values corresponding to “true” dimers, trimers, tetramers, pentamers and hexamers are marked in bold for comparison with the percentages in Table 1. The more negative the ΔG_rel_, the more stable the metallacycle (the monomer is not a metallacycle) while the higher the *δ*ΔG_rel_ the less stable the metallacycle for any given *n*.

To compare the data in Table 1 (crystal structures) and Table 5 (free energies) it is necessary to remember that in Table 1 the “2 + 2” and “3 + 3” structures are classified as dimers and trimers not as tetramers and hexamers, thus even if there are “3 + 3” structures that are more stable than hexamers, this does not affect the order of the values in bold.

Several main conclusions can be drawn from the values reported in Table 6:

1. Experimental metallacycles: mainly trimers, then dimers and tetramers, some hexamers, no pentamers.

2. Experimental cyclamers (NH-pyrazoles): dimers, tetramers and trimers, are common; hexamers are very rare and there are no pentamers. This is not identical but reasonably similar to the trend in experimental metallacycles. Note that the differences in cyclamers are insignificant (less than 6 kJ mol^−1^) compared with metallacycles (Cu: −211.4/−269.3; Ag: −171.6/−232.3; Au: −165.5/−269.3 kJ mol^−1^); this explains that steric effects of the substituents in cyclamers are sufficient to explain the size of the cycle (see point 6).

3. The absence of pentamers in the CSD can be due to the fact that pentameric species are crystallographically prohibited by “normal” rotational symmetry. Thus, perhaps more of these species exist but have not been crystallized for this reason.

4. Calculated metals: the order (in bold, Table 4) for Cu and Ag is hexamers, pentamers, tetramers, trimers and dimers; for Au the order is hexamers, tetramers, trimers, pentamers and dimers.

5. Metals: comparison of experimental vs. calculated values shows that there is no relationship between these, which means that the ring size is not the determining factor. Other factors such as steric effects of the substituents in the 3- and 5-positions, the roles of ancillary ligands, solvates and co-crystals as well as the kinetics of crystal growth could all play a determining role.

6. NH: there is a weak relationship for experimental vs. calculated values. Remember that in the experimental case the main factor is the steric effect of the substituents at the 3- and 5-positions [26].

7. There is no relationship between the order of calculated metals vs. that of calculated H.

### 2.4. QTAIM Analysis

This study was limited to Au(I) because, as explained in the introduction, it is the most interesting metal and we have published two significant papers on this topic [17,19]. The AIM analysis was also employed successfully in a related work [16].

**Figure 18:**
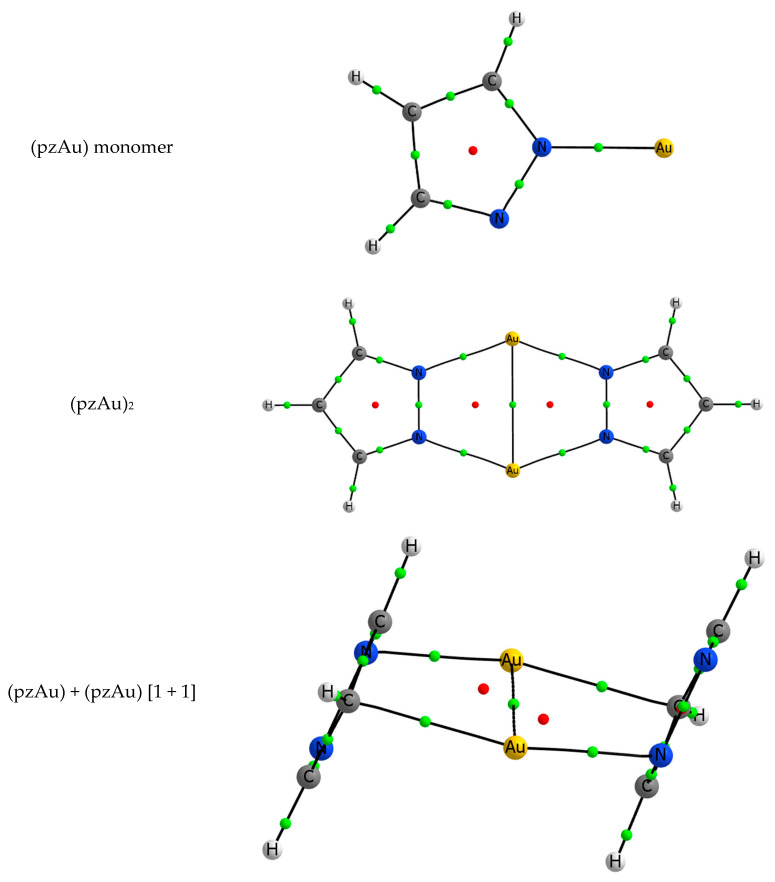

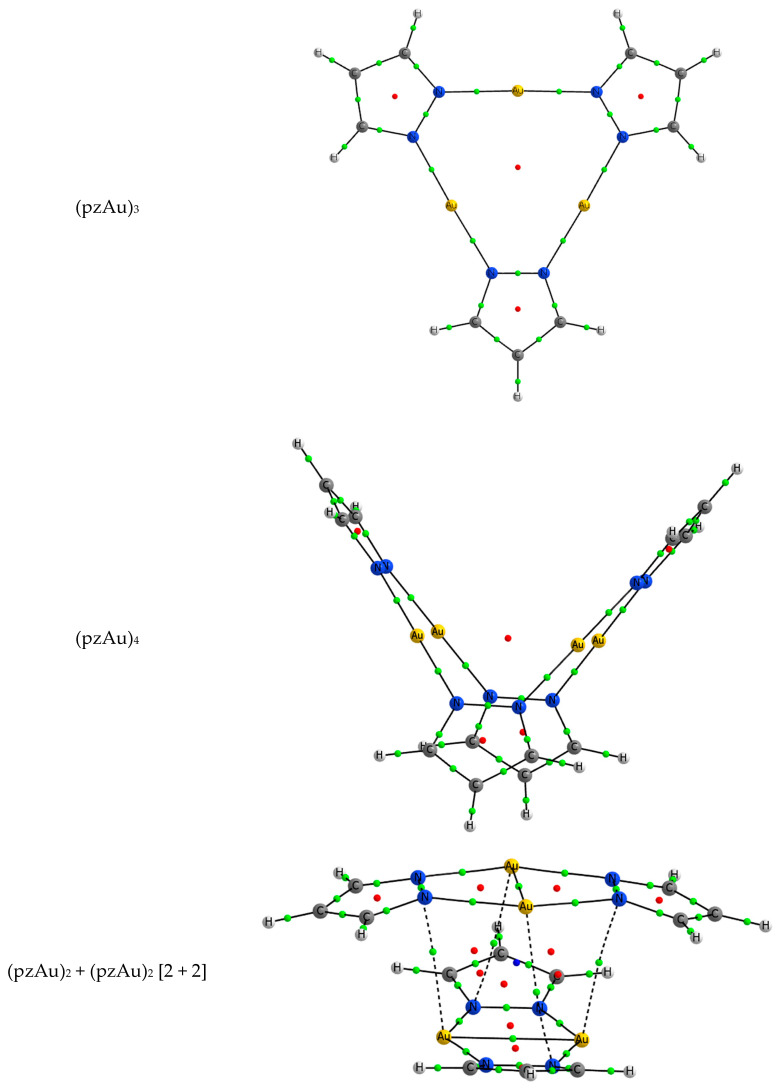

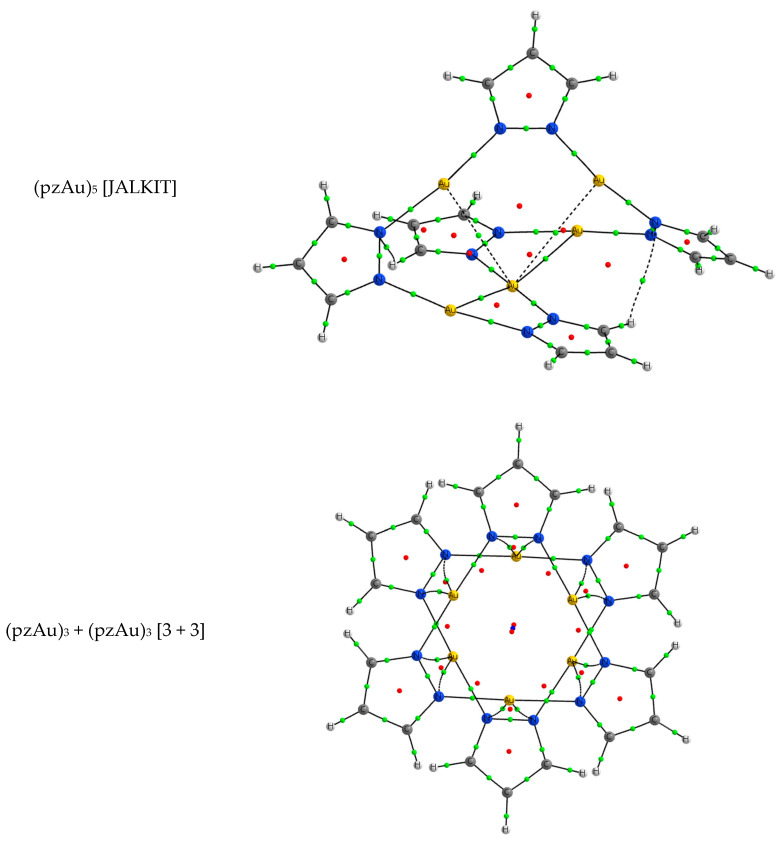

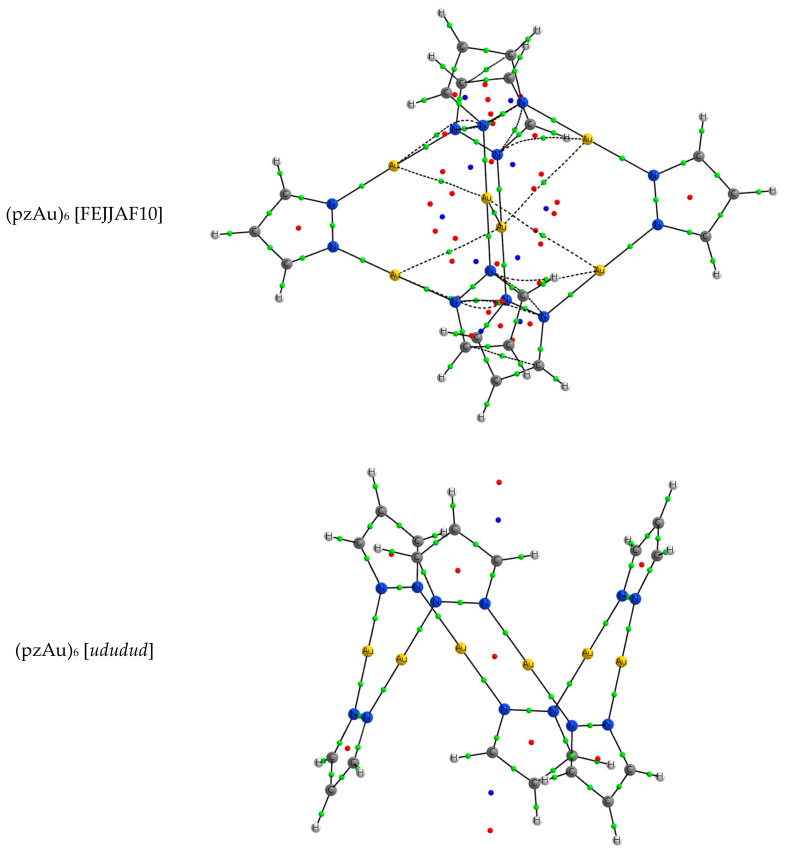
Molecular graph of the (pzAu)*_n_* complexes studied. The location of the bond, ring, and cage critical points are indicated with green red and blue dots.

Analysis of the electron density within the QTAIM shows the presence of bond critical points (BCPs) that link the gold atoms with the nitrogen atoms, other gold atoms, and in one case, with a carbon atom (1 + 1). The molecular graph of all of the systems is provided in Figure 18 with an indication of the position of the electron density critical points and the bond paths that link the BCPs with the nuclei. The topological description is very simple for the monomer, trimer, tetramer, and hexamer (*ududud*), in which only sequential Au-N BCPs are found linking the different systems. In the rest of the cases, an Au-Au BCP and additional Au-N BCPs are found. The Au-N BCPs (17 unique contacts) are found for interatomic distances between 1.99 and 3.68 Å. The electron density at the BCPs ranges between 0.145 and 0.006 au, thus showing in all cases positive Laplacian values (between 0.423 and 0.018 au). These results are characteristics of BCPs between atoms with very different electronegativities. The negative value of the total energy at the BCP for those contacts with interatomic distances shorter than 2.2 Å is an indication of the partial covalent character of these interactions.

The Au-Au BCPs (seven unique cases) are present for interatomic distances between 2.81 and 4.02 Å. The electron density values range between 0.042 and 0.005 au with positive Laplacian values. As observed previously, some of the BCPs present negative values for the total energy density (interatomic distances shorter than 3.4 Å).

In the two types of BCPs analyzed in this research, excellent exponential relationships (*R*^2^ > 0.99) were found between the electron density or the Laplacian at the BCP vs. the interatomic distance, a finding that it is consistent with previous reports in the literature for other contacts [16,74,75,76,77,78].

## 3. Methods

The crystal structures with (pzM)*_n_* systems were searched in the CSD database 5.41 (November 2019) [20]. The M06-2x DFT functional [79] in combination with the jul-cc-pVDZ basis set [80,81] for the light atoms (C, N and H) and the aug-cc-pVDZ-PP effective core potential basis set [82] for the Cu, Ag and Au atoms were used for the theoretical calculations, all of them for isolated molecules in gas phase. The geometry optimization and frequency calculations were carried out with the Gaussian-16 package [83]. In all cases, the geometries obtained correspond to energetic minima (no imaginary frequencies).

The electron density of the systems was analyzed within the quantum theory of the atoms in molecules (QTAIM) [21,23] theory with the AIMAll program [84]. This program allows location and characterization of the critical points of the electron density (nuclear attractor, bond, ring and cage critical points).

## 4. Conclusions

The main conclusions of this work concerning the structure in the solid state of metallacycles of pyrazolates and coinage metals are:

1. The exploration of the CSD yielded a considerable number of crystal structures and this allowed a statistical analysis of the abundance of different cycles.

2. All examples contain only a single metal although it should not present any difficulties to prepare metallacycles with two or three metals.

3. Dimers and trimers are common in the case of Cu(I). Dimers in all cases contain other ligands. Double trimers (3 + 3) should not be confused with hexamers, which are not known. Pentamers are also not known. There is no reason why hexamers could not be prepared, but the main difficulty is that a method does not exist that allows selection a priori of the size of the ring.

4. Dimers and trimers are also common in the case of Ag(I). There are examples in which the hexagonal ring of dimers is planar, folded (boat-type) and folded (chain-type). In this case there are examples of “true” hexamers (no “3 + 3” double trimers), but otherwise Ag(I) and Cu(I) are similar.

5. In the case of Au(I) dimers are not known “all dimers are Au(III) derivatives”. The double trimers form different patterns that can be classified according to the triangles formed by the three Au atoms. Tetramers are frequently found.

6. Calculations on simplified models (i.e., without C-substituents or other ligands) reproduce well the geometries but not the energies found experimentally, with stability increasing with ring size.

7. AIM analysis of the gold derivatives shows the presence of several Au-N and Au-Au BCPs and in one case an Au-C BCP.

## Figures and Tables

**Table 1:** Structures found in the Cambridge Structural Database (CSD) for metallacycles formed by pyrazolate ligands and the coinage metals M = Cu(I), Ag(I) and Au(I): (pzM)*_n_* for *n* = 2, 3, 4, 5 and 6. The percentages are in brackets. For comparative purposes, the results for NH-pyrazoles (cyclamers) are also provided. The relative order from frequent to zero (pentamers) is in bold.

Metal/H	Total	Dimers	Trimers	Tetramers	Pentamers	Hexamers (Refcodes)
Cu(I)	81	**2** 22 [27.2]	**1** 41 [50.6]	**3** 18 [22.2]	**5** 0 [0.0]	**4** 0 [0.0]
Ag(I)	96	**2** 6 [6.2]	**1** 72 [75.0]	**3** 6 [6.2]	**5** 0 [0.0]	**4** 2 (QEJJEX [54], QEJJIB [54]) [2.1]
Au(I)	37	**4** 0 [0.0]	**1** 28 [75.7]	**2** 8 [21.6]	**5** 0 [0.0]	**3** 1 (FEJJAF10 [62] [2.7]
H [26]	38	**1** 16 [42.1]	**3** 8 [21.1]	**2** 13 [34.2]	**5** 0 [0.0]	**4** 1 [2.6]

**Table 2:** Calculated mean metal···metal and metal···N atom (Å) (averaged and parent pyrazoles).

Metal	Dimer		Trimer		Tetramer		Hexamer	
	M-M	N-M	M-M	N-M	M-M	N-M	M-M	N-M
Cu(I)	2.656	1.963	3.306	1.921	3.352	1.920	3.447	1.932
Ag(I)	2.953	2.192	3.507	2.124	3.448	2.122	3.631	2.145
Au(I)	2.808	2.124	3.440	2.041	3.478	2.037	3.605	2.042

**Table 3:** Measured mean metal···metal and metal···N atom (Å) (averaged and parent pyrazoles).

Metal	Dimer (With Ligands)		Trimer		Tetramer		Hexamer	
	M-M	N-M	M-M	N-M	M-M	N-M	M-M	N-M
Cu(I)	3.424 ^a^	1.974 ^a^	3.228 ^c^	1.858 ^c^	3.095 (3)	1.849 (3)	-	-
Ag(I)	3.824 ^b^	2.284 ^b^	3.520 ^d^	2.090 ^d^	3.274 (5)	2.073 (5)	3.615 (5)	2.084 (5)
Au(I)	-	-	3.356 ^e^	1.994 ^e^	3.185 (9)	2.006 (5)	3.121 (10)	2.059 (10)

**Experimental (averaged values)** (the compounds used corresponds to those represented in the corresponding Figures in blue) or to compounds (refcodes) cited in the discussion. Some have been selected to build up this Table. ^a^ BITSAB [63], IPIGET [35], JEMCAF [32], NETMAD [37]; ^b^ FINWIL [64], KIRXIV [65], ZIGROZ [66], ZIGRUF [67]; ^c^ BELTOC [42], CODBAB [40], VIJMUW [41], XELXAN [43]; ^d^ AWAWUS [67], CENFIM [68], HICHIL [69], XOGJUA [45]; ^e^ COHFIO01 [50], FUWXOK01 [58].

**Table 4:** Experimental metal···metal and metal···N atom (Å) (averaged and parent pyrazoles).

Metal	Dimer (With Ligands)		Trimer		Tetramer		Hexamer	
	M-M	N-M	M-M	N-M	M-M	N-M	M-M	N-M
Cu(I)	3.726 ^a^	2.010 ^a^	3.251 ^c^	1.861 ^c^	3.394 ^f^	1.962 ^f^	-	-
Ag(I)	3.788 ^b^	2.245 ^b^	3.426 ^d^	2.200 ^d^	None	None	None	None
Au(I)	-	-	3.382 ^e^	2.004 ^e^	None	None	None	None

**Experimental** (unsubstituted pyrazoles or, at least, only 4-substituted pyrazoles). When neither HHH or HRH pyrazoles were found, none are written in the Table.^a^ KIRXOB [65], NETLUW [37]; ^b^ RATFAT [70], RATFEX [70]; ^c^ No example with the parent pyrazole, instead the 4-chloro derivative (CODBAB [40]) was used; ^d^ HESBUC [4]; ^e^ MUTKUH [59]; ^f^ No example with the parent pyrazole, instead the 4-*n*-butyl derivative (FORGIE [71]) was used.

**Table 5:** Relative free energies per monomer, ΔG_rel_ and *δ*ΔG_rel_, in kJ mol^−1^, of the metallacycles formed by the parent pyrazolate ligand and the coinage metals M = Cu(I), Ag(I) and Au(I): (pzM)*_n_* for *n* = 2, 3, 4, 5 and 6.

	Cu(I)		Ag(I)		Au(I)		H	
*n*-mer	ΔG_rel_	*δ*ΔG_rel_	ΔG_rel_	*δ*ΔG_rel_	ΔG_rel_	*δ*ΔG_rel_	ΔG_rel_	*δ*ΔG_rel_
Monomer	0	-	0	-	0	-	0	-
**Dimer**	**−211.4**	**0.0**	**−171.6**	**0.0**	**−165.5**	**0.0**	**−3.5**	-
1 + 1	−149.2	124.3	−126.4	90.4	−108.0	115.0	-	-
**Trimer**	**−254.4**	-	**−213.0**	-	**−256.0**	-	**−4.5**	-
**Tetramer**	**−256.0**	**0.0**	**−214.2**	**0.0**	**−257.8**	**0.0**	**−5.6**	-
2 + 2	−235.0	84.3	−202.8	45.4	−182.4	301.6	-	-
2 + 2 twisted	−229.4	106.7	−193.2	83.6	−182.1	302.8	-	-
**Pentamer**	**−259.4**	-	**−218.5**	-	**−253.9**	-	-	-
**Hexamer**	**−269.3**	**31.3**	**−230.0**	**33.5**	**−269.3**	**21.6**	**−2.0**	**10.6**
Hexamer *ududud*	−255.4	114.6	−213.6	131.9	−257.3	93.6	−3.7	0.0
3 + 3	−274.5	0.0	−235.6	0.0	−272.9	0.0	-	-
3 + 3 twisted	−271.3	19.4	−232.2	20.0	−272.6	1.8	-	-

**Table 6:** Comparison of the populations of Table 1 and Table 5 (absolute values).

Metal/H	% Dimers	% Trimers	% Tetramers	% Pentamers	% Hexamers
Cu(I)	**2** [27.2]	**1** [50.6]	**3** [22.2]	**5** [0.0]	**4** [0.0]
Ag(I)	**2** [6.2]	**1** [75.0]	**3** [6.2]	**5** [0.0]	**4** [2.1]
Au(I)	**4** [0.0]	**1** [75.7]	**2** [21.6]	**5** [0.0]	**3** [2.7]
H	**1** [42.1]	**3** [21.1]	**2** [34.2]	**5** [0.0]	**4** [2.6]
	ΔG_rel_ dimers	ΔG_rel_ trimers	ΔG_rel_ tetramers	ΔG_rel_ pentamers	ΔG_rel_ hexamers
Cu(I)	**5** 211.4	**4** 254.4	**3** 256.0	**2** 259.4	**1** 269.3
Ag(I)	**5** 171.6	**4** 213.0	**3** 214.2	**2** 218.5	**1** 232.2
Au(I)	**5** 165.5	**3** 256.0	**2** 257.8	**4** 253.9	**1** 269.3
H	**4** 3.5	**2** 4.5	**1** 5.6	-	**3** 3.7 **5** 2.0
